# Novel Circovirus from Mink, China

**DOI:** 10.3201/eid2009.140015

**Published:** 2014-09

**Authors:** Hai Lian, Ye Liu, Nan Li, Yuying Wang, Shoufeng Zhang, Rongliang Hu

**Affiliations:** Academy of Military Medical Sciences, Changchun, China

**Keywords:** Circovirus, mink, diarrhea, China, viruses

## Abstract

A long-established epidemic of enteritis, caused by an unidentified pathogen distinct from parvovirus, has now been recognized in mink. In 2013, we identified a novel circovirus by degenerate PCR and fully sequenced its genome. This virus differs substantially from currently known members of the genus *Circovirus* and represents a new species.

Members of the family *Circoviridae* are nonenveloped, icosahedral viruses with diameters of 16–26 nm and a small, circular, single-stranded DNA genome (the smallest known autonomously replicating viral genome) (*1*). The family *Circoviridae* is currently composed of 2 genera, *Gyrovirus* and *Circovirus*, each of which has been associated with diseases in birds, pigs, and dogs (*1*,*2*). Among birds, circovirus infections have been associated with beak abnormalities, lethargy, and anorexia in parrots and pigeons and production losses and death in geese (*3*,*4*). Among pigs, porcine circovirus type 2 (PCV-2) causes respiratory and enteric diseases, dermatitis, and reproductive problems, resulting in major economic losses to the pork industry (*5*,*6*). A proposed new genus (*Cyclovirus*) within the family *Circoviridae* consists of cycloviruses. Genomes of cycloviruses have been identified in human and chimpanzee feces and human cerebrospinal fluid (*7*,*8*). Recently, circoviruses have been found in bats and have shown high genetic diversity (*9*,*10*). We describe a novel circovirus in mink (mustelids). This mink circovirus (MiCV) was found in the liver, digestive tract, and fecal specimens from mink with diarrhea as their main clinic sign.

Mink enteritis is caused by a parvovirus (*11*). However, around 1978, on some mink farms in Dalian, Liaoning Province, China, ≈7% of mink that had received the inactivated parvovirus vaccine had diarrhea and anorexia that resulted in death. The farmers called this refractory diarrhea. According to the observations of the farmers, the incubation period of this disease was <10 days. The preliminary clinical signs included lethargy, anorexia, pale muzzle, and unkempt fur. The feces were initially white and then became jelly red or yellow. Several days after the appearance of red or yellow feces, ≈7% of affected mink died. All mink from farms with disease seemed to have been affected; 70%–80% showed clinical signs, but most recovered.

Within a few years of farmers first noticing the disease, a major outbreak occurred during 1985–1990 in Dalian and on mink farms in the surrounding mountain regions. The mink in this area are pedigree breeds with premium quality fur; all other farms introducing stock from this area were also affected. Spread of the infection seemed to be horizontal because mink were usually affected after a mink on the same farm became ill. However, the epidemic now seems to be limited, potentially in part because of the practice of autogenous vaccination that uses formalin-inactivated supernatant from tissue suspension from affected minks.

## The Study

During September–October 2013, we collected liver, digestive tract (gut), and fecal samples from 43 mink (26 with diarrhea and 17 healthy) ≈6–10 months of age from 3 mink farms in Dalian, China. Using PCR-based methods, we excluded the following as causative agent: mink enteritis virus, canine distemper virus, Aleutian mink disease virus, mink orthoreovirus, and mink coronavirus. To identify the causative agent, we separately homogenized fecal, liver, and gut samples from diseased unvaccinated mink in phosphate-buffered saline and then submitted the supernatants to negative staining and observation under an electron microscope. We found circovirus-like particles, but not in typical lattice arrangement, in liver and gut samples. We performed PCRs with degenerate primers (CV-F1, CV-R1, CV-F2, and CV-R2; Table 1) based on highly conserved amino acid motifs in the Rep proteins of circoviruses and cycloviruses. Products of ≈400 bp were purified and sequenced by using primer CV-R2. 

**Table 1:** Oligonucleotide sequences of primers used in study of novel circovirus isolated from mink, Dalian, China

Primer	Oligonucleotide sequence, 5′→3′	Reference.
CV-F1	GGIAYICCICAYYTICARGG	(7)
CV-R1	AWCCAICCRTARAARTCRTC	(7)
CV-F2	GGIAYICCI CAYYTICARGGITT	(7)
CV-R2	TGYTGYTCRTAICCRTCCCACCA	(7)
CV-F3	GCCCGCTTAAACGGCTCAAACCGCATTTTC	Designed for this study
CV-R3	TGGGAGGGGCCTGAGGGATTACGTCATACA	Designed for this study
CV-F4	GCAGTAAGTCTCCCCCTTTACTGCAATATC	Designed for this study
CV-R4	CTTGCTGAATAATGGCGGAACAATGACTGA	Designed for this study

All samples from mink with diarrhea were positive by PCR for the same circovirus-like Rep sequence; all samples from healthy mink were negative (Table 2), showing a strong relationship between the identified virus and disease. A BLAST search (http://www.ncbi.nlm.nih.gov/blast/Blast.cgi) showed the sequence to be an authentic circovirus sequence, with closest similarity (73%) to the bat circavirus (BtCV) from the *Rhinolophus ferrumequinum* group XO bat (XOR) genome, recently identified in bats by metagenomic analysis of tissue samples (*10*).

**Table 2:** Prevalence of mink circovirus DNA in mink, Dalian, China

Specimen tested	No. mink	Age of mink, mo. (no. mink)	Farm*	Positive for mink circovirus
Mink with diarrhea				
Liver	12	6–7 (8), 7–8 (3), 9–10 (1)	4 from farm 1, 5 from farm 2, 3 from farm 3	12
Gut	12	6–7 (8), 7–8 (3), 9–10 (1)	4 from farm 1, 5 from farm 2, 3 from farm 3	12
Feces	19	6–7 (12), 7–8 (5), 9–10 (2)	7 from farm 1, 8 from farm 2, 4 from farm 3	19
Healthy mink				
Liver	9	6–7 (6), 7–8 (3)	4 from farm 1, 3 from farm 2, 2 from farm 3	0
Gut	9	6–7 (6), 7–8 (3)	4 from farm 1, 3 from farm 2, 2 from farm 3	0
Feces	11	6–7 (7), 7–8 (3), 9–10 (1)	4 from farm 1, 5 from farm 2, 2 from farm 3	0

*Farms 1 and 2 located in Zhuanghe County, Dalian, China; farm 3 located in Pulandian County, Dalian

.

To obtain the complete genome sequence of MiCV, we used primers CV-F3, CV-R3, CV-F4, and CV-R4 (Table 1) for inverse PCR, together with Premix PrimeSTAR HS DNA polymerase (Takara Bio, Inc., Dalian, China). The product was sequenced in duplicate. The complete circular genome of MiCV-DL13 contained 1,753 nt (GenBank accession no. KJ020099); highest similarity was 64.1% with the genome of BtCV-XOR. The putative Rep protein of MiCV-DL13 was 297 aa, and it shared 50.7%, 51.6%, 55.4%, and 79.7% aa identity with the Rep protein of pig (PCV2 AUT1, AY424401), bird (GoCV, AJ304456), dog (DogCV-1698, NC_020904) (*2*), and bat (BtCV-XOR, JX863737), respectively, circoviruses. The results of phylogenetic analyses based on the amino acid sequence of the Rep protein are shown in the Figure. The deduced capsid protein is 227 aa; highest similarity is 47.3% with porcine circovirus 2 (EU-RO-WB2006–38, JN382157) from wild boars in Romania (*12*). 

**Figure:**
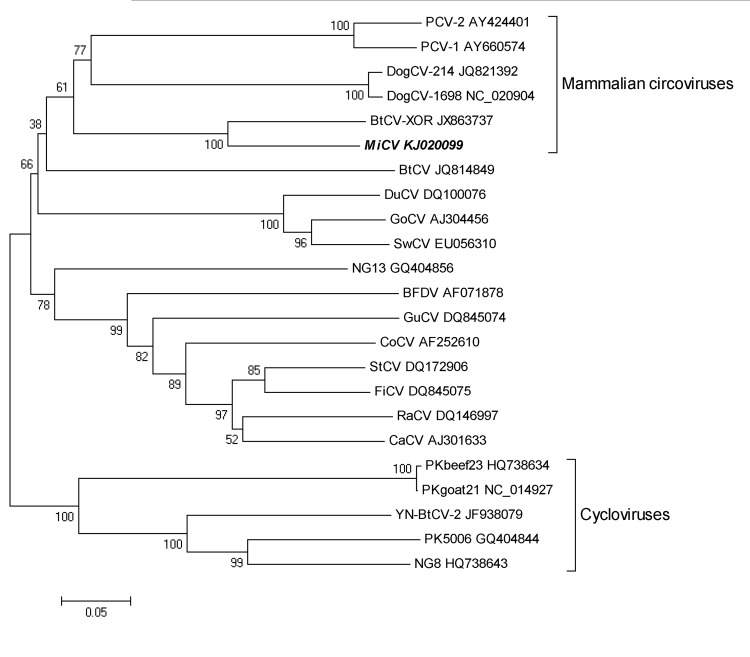
Phylogenetic tree constructed on the basis of the Rep protein sequence of the mink circovirus by using the neighbor-joining method in MEGA5 (http://www.megasoftware.net). Representative members of the genera *Circovirus* and *Cyclovirus* were included in the analysis, and GenBank accession numbers are indicated. Numbers at nodes indicate bootstrap values based on 1,000 replicates. Scale bar indicates nucleotide substitutions per site. The strain sequenced from the mink in Dalian, China, during 2013 (this study) is indicated in boldface italics**.** PCV-2, porcine circovirus type 2, PCV-1, porcine circovirus type 1, DogCV, dog circovirus; BtCV, bat circovirus; MiCV, mink circovirus; DuCV, duck circovirus; GoCV, goose circovirus; SwCV,swan circovirus; NG13, human stool-associated circular virus NG13; BFDV, beak and feather disease virus; GuCV,gull circovirus; CoCV, columbid circovirus; StCV, starling circovirus; FiCV, finch circovirus; RaCV, raven circovirus; CaCV, canary circovirus; PKbeef23, cyclovirus PKbeef23/PAK/2009; PKgoat21, cyclovirus PKgoat21/PAK/2009; PK 5006, cyclovirus PK5006; NG8, cyclovirus NGchicken8/NGA/2009.

The International Committee for the Taxonomy of Viruses has suggested criteria for circovirus species demarcation: genome nucleotide identities of <75% and capsid protein amino acid identities of <70% (*1*). The MiCV reported here therefore represents a new circovirus species.

## Conclusions

Apart from the well-documented pathogenicity of porcine circovirus in pigs, of dog circovirus in dogs, and of other circoviruses in birds, the biological significance of widespread cyclovirus, circovirus, and circovirus-like virus infections in other domesticated and wild animals remains unknown. In this study, the novel circovirus, MiCV, was identified in liver and digestive tract samples from mink with diarrhea. No MiCV DNA was found in any healthy mink, thereby indicating a strong relationship between the isolated virus and disease. Whether other mustelids (e.g., weasels, badgers, and ferrets) or even humans in close contact with infected mink are susceptible to this virus merits further study.
